# Curcumin nanoparticles incorporated in PVA/collagen composite films promote wound healing

**DOI:** 10.1080/10717544.2020.1853280

**Published:** 2020-11-30

**Authors:** QingQing Leng, Yue Li, XianLun Pang, BiQiong Wang, ZhouXue Wu, Yun Lu, Kang Xiong, Ling Zhao, Ping Zhou, ShaoZhi Fu

**Affiliations:** a Department of Oncology, The Affiliated Hospital of Southwest Medical University, Luzhou, China; b Health Management Center, Traditional Chinese Medicine Hospital, Southwest Medical University, Luzhou, China; c Department of Pharmaceutics, School of Pharmacy of Southwest Medical University, Luzhou, China; d Department of Radiology, The Affiliated Hospital of Southwest Medical University, Luzhou, China

**Keywords:** Curcumin, nanoparticles, collagen, PVA, composite film, wound healing

## Abstract

Skin repair remains a common problem in plastic surgery. Wound dressing plays an important role in promoting local skin healing and has been widely studied. This study aimed to manufacture a composite film (CPCF) containing curcumin nanoparticles, collagen, and polyvinyl alcohol (PVA) to effectively promote the healing of skin wounds. Sustained drug release from the composite film provides long-term protection and treatment for skin wounds. Both antibacterial property and good histocompatibility of the CPCF were examined by analyzing antibacterial activity and cytotoxicity to validate its applicability for wound management. Moreover, *in vivo* studies proved that the CPCF had a rapid healing rate of 98.03%±0.79% and mature epithelialization on day 15 after surgery. Obvious hair follicles and earlier re-epithelialization was also noticed in the CPCF group using H&E staining. The result of Masson’s trichrome staining confirmed that CPCF could promote the formation of collagen fibers. In summary, CPCF may be promising as a wound dressing agent in wound management owing to its rapid wound-healing effects.

## 1. Introduction

The skin is the largest organ of the human body and performs the functions of protecting the body from damage, excessive water excretion, regulating body temperature, and perceiving external stimuli. A complete skin is vital to human health (Niranjana et al., 2019). The skin may be wounded by bruising, scratching or during surgery, as well as in disease conditions such as diabetes. Irrespective of the wound being acute or chronic, it may cause infection, loss of body fluids, electrolytes, and nutrients, which may severely threaten the quality of life and health of individuals (Xu et al., 2015; Zhao et al., 2017). Therefore, it is very important to promote wound healing and restore skin function by adopting various treatment measures clinically.

Among several available treatment strategies, covering wounds with dressing to promote skin healing is a popular method mostly used by clinicians. To promote wound healing, the dressing material should not only be convenient to use, but should also possess hemostatic and anti-infective properties and exhibit histocompatibility. Additionally, it should be capable of reducing wound-healing time, the side effects of drugs and improving bioavailability (Mohammadi et al., 2019). With the development of material science and technology, many novel wound dressings, such as injectable hydrogels with photothermal antibacterial ability (Liang et al., 2020; Huang et al., 2020), cellulose sponges (Ye et al., 2018), nanofibers (Dhand et al., 2017), and films (Shanmugapriya et al., 2018), have been designed to promote wound healing. Films with a homogeneous polymeric structure have played a significant role in preventing microbial infection at the wound surface in recent years (Shanmugapriya et al., 2018). Incorporating therapeutic drugs into these films increases the therapeutic effect of drugs without altering the film function would be a good idea in skin tissue engineering.

Wound exposure often causes bacterial infections and inflammation, which further affects wound healing. Therefore, if anti-inflammatory and antibacterial drugs can be incorporated into wound dressings, they would be of great benefit for skin repair. For instance, Zoheyr Mohammadi produced chrysin-curcumin-loaded nanofibers to promote wound healing (Mohammadi et al., 2019). Curcumin, a naturally occurring small-molecule compound, has anti-inflammatory and antibacterial properties among other biological activities (Guo et al., 2011). However, its low water solubility restricts its clinical use (Choudhury et al., 2016). Our previous study shows that curcumin loaded poly(ε-caprolactone)-poly(ethylene glycol)-poly (ε-caprolactone) (Cur/PCEC) nanofibers can increase the bioavailability and stability of curcumin hence enhance wound healing (Fu et al., 2014). Therefore, incorporating curcumin into composite films to fabricate a skin-dressing product would serve as a beneficial and viable approach to study the effect of curcumin on wound healing.

As a skin substitute, an ideal wound dressing should mimic skin composition, structure and accelerate the process of skin repair. Collagen, a component of skin, is the most abundant and widely distributed functional protein in mammals. It has the advantages of low antigenicity and low inflammatory response and is known for its biodegradable properties. It is an essential component required for the highly ordered structural integrity and tensile strength of the skin (Suh et al., 2018; Kang et al., 2019). Collagen-based biomaterials with different structures have been used for tissue repair and regeneration (Kenar et al., 2019; Gil-Cifuentes et al., 2019). However, the use of collagen alone has some disadvantages such as difficulty in molding and it is fragile. To address these problems, collagen is often incorporated into other materials such as chitosan, poly(L-lactic acid) (PLLA) polymer, and polyvinyl alcohol (PVA) to enhance the formation of a composite film (Socrates et al., 2019; Liang et al., 2019). Among these, PVA gains widespread attention, since it is water soluble, nontoxic, noncarcinogenic, biodegradable, biocompatible, transparent, and with high charge storage ability, along with superior film-forming attributes (Chen et al., 2020; Qiu et al., 2020). Chetouani et al designed a biocompatible and antimicrobial film based on CS/oxidized pectin/PVA copolymer, which displayed high swelling ratio and good mechanical properties, exhibiting its potential as a wound dressing (Chetouani et al., 2017). In this study, we blended collagen type I, PVA, and antibacterial drugs (Cur/PCEC nanoparticles) to construct a composite film which is used for wound dressing.

**Scheme 1.**
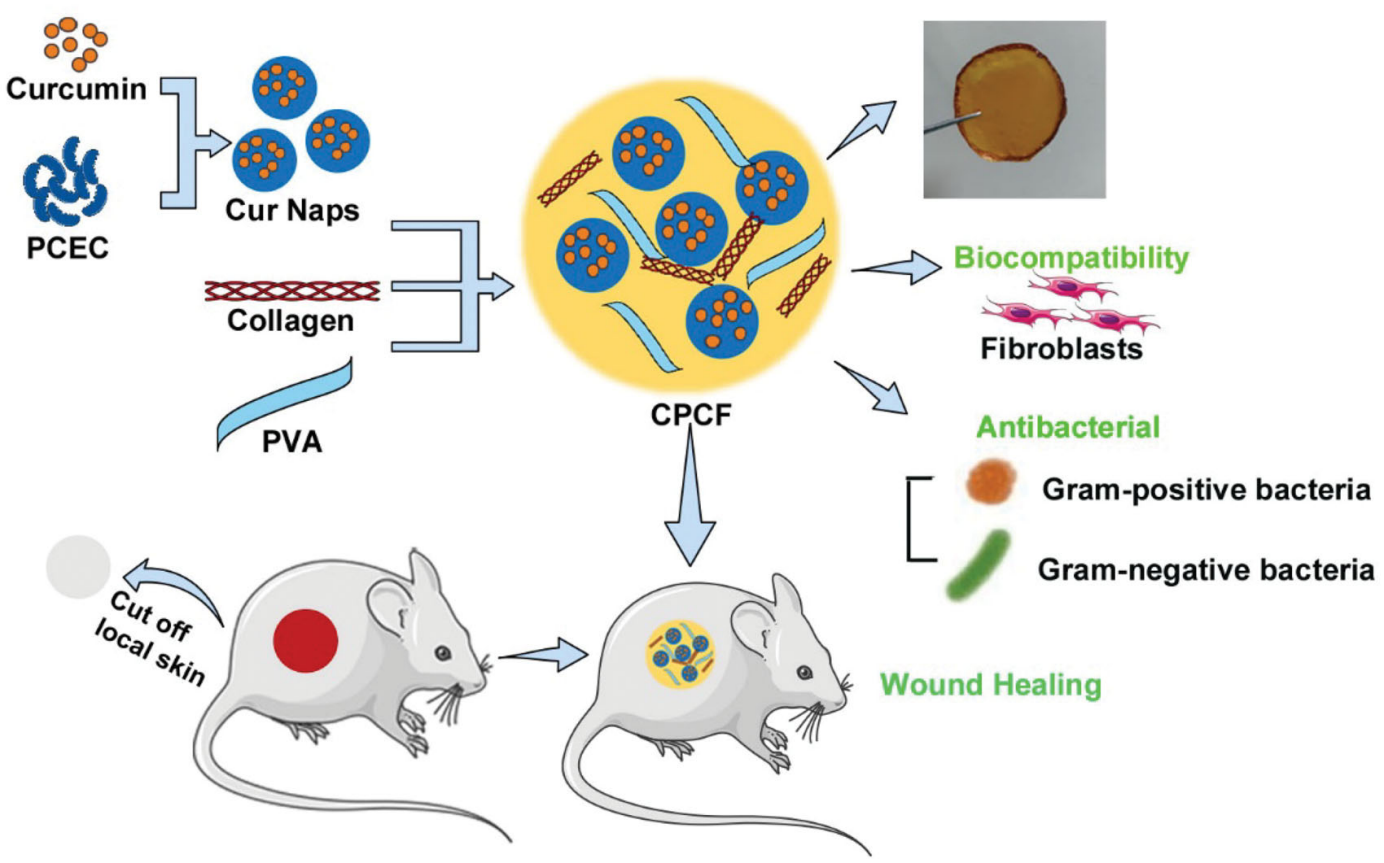
Schematic illustration of preparation process of curcumin nanoparticles incorporated PVA/Collagen composite films (CPCF) and the application for wound treatment.

We fabricated a PVA/collagen composite film containing Cur/PCEC nanoparticles for promoting wound healing as indicated in Scheme 1. First, the PCEC polymer was used as a carrier to deliver curcumin by forming curcumin nanoparticles. Then the Cur/PCEC nanoparticles were incorporated with collagen and PVA to build a composite film used as a wound dressing for skin. In addition, we evaluated *in vitro* drug release and cytotoxicity by HPLC and MTT assay, respectively. The antibacterial property of the composite film (CPCF) was assessed using drug allergy testing. Lastly, we established a skin trauma model using Sprague-Dawley rats to evaluate the therapeutic effects of the CPCF on skin wounds *in vivo.*

## 2. Materials and methods

### 2.1. Materials

Curcumin was purchased from Aladdin Industrial Co. (Shanghai, China). Poly(ε-caprolactone)-poly (ethylene glycol)-poly(ε-caprolactone) (PCL-PEG-PCL, PCEC, MW = 3700) was prepared in our laboratory. Collagen type I was purchased from Solarbio Science & Technology Co. Ltd. (Beijing, China). PVA was purchased from Sigma Aldrich (St. Louis, MO, USA). Dichloromethane and Methyl alcohol were purchased from Chengdu Kelong Co. Ltd. (Chengdu, China). Dichloromethane and methyl alcohol were of analytical grade and used directly without further purification. *Staphylococcus aureus* ATCC29213 and *Escherichia coli* OP50 were obtained from the Microbiology Laboratory, School of Basic Medicine, Southwest Medical University. Male SD rats weighting 220–260 g (8 weeks of age) were purchased from Chengdu Dashuo Experimental Animal Co. Ltd. (Chengdu, China).

### 2.2. Preparation of cur/PCEC nanoparticles and cur/PVA/collagen composite films (CPCF)

In this study, PCL-PEG-PCL (PCEC) copolymer was compounded by ring-opening polymerization of ε-CL and PEG (Mn = 2000) catalyzed by Sn (Oct)_2_ at 135 °C. Then, it was purified and vacuum dried to a constant weight according to a previously published report (Wang et al., 2011).

We used PCEC as a carrier to prepare curcumin-load Cur/PCEC nanoparticles. Briefly, 20 mg curcumin powder was dissolved in methyl alcohol and 180 mg PCEC copolymer was completely dissolved in dichloromethane. The two solutions were transferred to round bottom flask and mixed completely. Next, the solvents were removed by rotary evaporation at 37 °C. Then, the thin film on the wall of the round bottom flask was reconstituted using deionized water at 60 °C and the resulting solution was filtered through a 220 nm filter to obtain a clear solution. Lastly, the solution was dried in vacuum below −50 °C for 72 h to obtain the Cur/PCEC powder. To prepare the CPCF (Cur/PVA/Collagen ccomposite film), 60 mg PVA was dissolved in 2 mL double distilled water using magnetic stirring at 60 °C and then cooled to around 25 °C. Next, 30 mg of collagen type I and 30 mg dried Cur/PCEC powder were added to the PVA solution and stirred well. Lastly, the mixed solution was poured into a circular mold and left in a laminar flow dryer at 45 °C for three hours to form the composite film.

### 2.3. Characteristics of cur/PCEC nanoparticles

The particle size and polydispersity index (PDI) of Cur/PCEC nanoparticles were evaluated three times using dynamic light scattering (NanoBrook90 plus Zeta, Brookhaven, NY, USA) at around 25 °C. Drug loading (DL) and encapsulation efficiency (EE) were determined using high-performance liquid chromatography (HPLC, Agilent 1260, Agilent Technologies, USA). The analytical column was a reversed-phase C18 alkyl silane column (150 mm × 4.6 mm, 5 μm, Agilent Santa Clara, CA) at 28 °C. The detection wavelength was set at 425 nm with a flow rate of 1.0 mL/min and an injection volume of 20 μL. The mobile phase consisted of 3% glacial acetic acid: methanol (40:60, v/v). The values are averages of three replicates. DL and EE were calculated using the following equations:

DL%=Drug/(Polymer+Drug)×100%

EE%= Actual DL/Theoretical DL×100%

The morphology of the Cur/PCEC nanoparticles was imaged by using transmission electron microscopy (TEM; JEM-1200EX, JEOL, Japan) at an accelerating voltage of 120 kV.

### 2.4. *In vitro* drug release

To explore the *in vitro* drug release behavior of curcumin from free curcumin, Cur/PCEC nanoparticles and CPCF, 2.88 mg free curcumin, 30 mg Cur/PCEC nanoparticles (containing 2.88 mg curcumin) or one piece of CPCF (containing 2.88 mg curcumin) were placed in dialysis bags (molecular weight cutoff, 3.5 kDa). The dialysis bags were immersed into 40 mL phosphate-buffered saline (PBS) containing Tween 80 (0.5%, w/v)) and incubated at 37 °C with mild shaking at a speed of 100 rpm. At regular time intervals (1, 3, 6, 9, 12, 24, 48, 72, 96, and 120 h), 2 mL of release medium was withdrawn and a similar volume of fresh pre-heated release medium was replaced. Samples were collected for HPLC and each measurement was conducted at least three times.

### 2.5. Anti-bacterial activity

In order to examine the antibacterial effect of CPCF prepared in this study, the antibacterial activity of samples was carried out using the Kirby-Bauer radial disk diffusion method against Gram-positive (*S. aureus* ATCC29213) and Gram-negative (*E. coli* OP50) bacteria. To determine antibacterial activity, LB nutrient agar medium and nutrient broth medium were formulated and sterilized. The LB nutrient agar media was poured into autoclaved/sterilized Petri dishes. Next, the bacterial strains were spread on nutrient agar and incubated at 37 °C for 24 h to obtain a single community. Representative bacterial colonies were picked using a coating ring placed in pre-sterilized nutrient broth. It was shaken at 37 °C for 2–8 h and diluted with an appropriate amount of nutrient broth to cultivate about 10^5^CFU/mL bacteria. Then, the prepared bacterial culture medium was spread evenly on the surface of the agar medium. A blank drug-sensitive paper measuring 6 mm × 6 mm, which was immersed in physiological saline, free curcumin solution, curcumin/PCEC nano solution, or stuck to PVA/Col film or CPCF, was placed on the center of the nutrient agar plate. Lastly, all plates were incubated at 37 °C for 12 h and the inhibitory ring around the drug-sensitive paper was measured to determine antibacterial activity.

### 2.6. *In vitro* cytotoxicity

*In vitro* cytotoxicity of free curcumin, Cur/PCEC nanoparticles, CPCF, and PVA/Col film on human skin fibroblasts were evaluated using the MTT assay. Sample solution of free curcumin, Cur/PCEC nanoparticles, CPCF, and PVA/Col film were prepared with different curcumin or collagen concentrations (1.25, 2.5, 5, 10, 20, 40 µg/mL). The cells were seeded at a density of 5 × 10^3^ cells/well in 96-well plates and cultured for 24 h. Experiments were initiated by replacing the culture medium in each well with 100 µL of sample solutions and cultivated at 37 °C in a CO_2_ incubator. After 48 h of incubation, 20 µL MTT reagent (5 mg/mL in PBS) was added to each well. The plates were incubated at 37 °C for a further 4 h. At the end of the incubation period, the medium was removed and 150 µL DMSO was added to each well and shaken for 10 min. Lastly, cell viability was analyzed and recorded at 490 nm using a microplate reader (iMark, USA). Medium-treated cells served as controls and the percentage of viable cells was calculated based on the absorbance values.

### 2.7. *In vivo* wound healing study

Male SD rats (8 weeks of age, weighing 220–260 g) were housed in cages in a special animal room and raised with water and commercial feed. A total of 28 rats were separated into four groups (*n* = 7) for *in vivo* experiments. Animal experiments were performed according to the animal care guidelines and were approved by the Institutional Animal Care and Treatment Committee of Southwest Medical University (Luzhou, China) (Approval number: 201906106). The wounds in animals in group 1 were untreated and used as a control; animals in group 2 were treated with aqueous Cur/PCEC NPs solution; and wounds of the animals in groups 3 and 4 were covered using PVA/collagen films and the CPCF, respectively. Before modeling, each animal was anesthetized using a hypodermic injection of 0.3 mL/100 g of 10% chloral hydrate. Then, a circular surgical area was created by removing the dorsal hair of mice. Subsequently, a full-thickness wound with a diameter of 2.5 cm was built on the circular surgical area. The size of the wound was measured and photographed on days 3, 6, 9, 12, and 15 after treatment. After the observation period, skin tissues at the wound sites were excised for histological examination.

## 3. Results

### 3.1. Preparation of cur/PCEC nanoparticles and cur/PVA/collagen composite films (CPCF)

**Figure 1.**
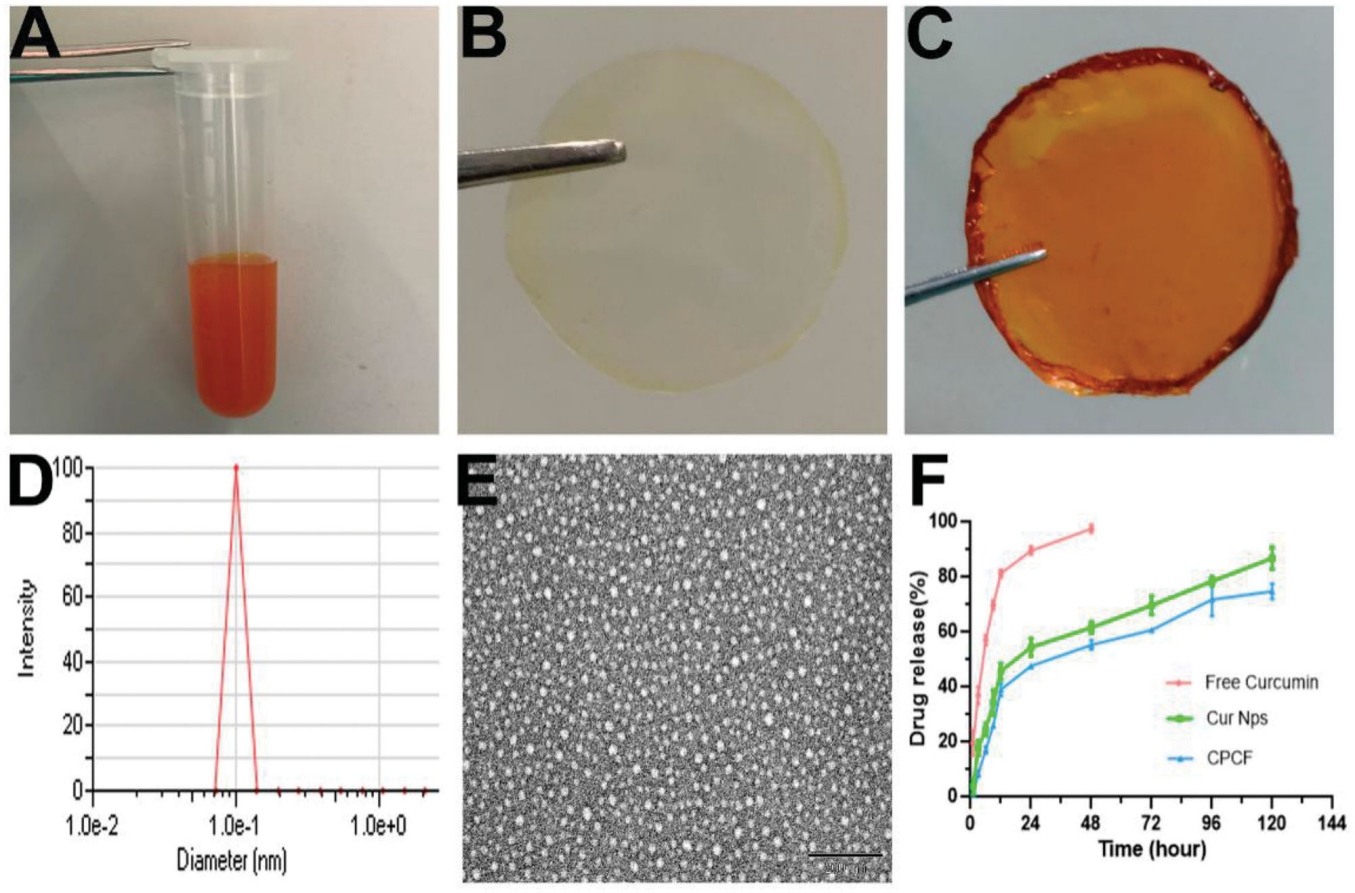
Photos of different samples used to treat wound defects: (A) aqueous Cur/PCEC nanoparticles, (B) PVA/Col film, (C) CPCF; And the particle size analysis (D), TEM image (E) of the Cur/PCEC nanoparticles; (F) the drug release of curcumin from free curcumin, Cur/PCEC Nps and CPCF.

In this study, we first loaded curcumin into the PCEC carrier to prepare Cur/PCEC nanoparticles (Figure 1(A)) and then combined it with collagen and PVA to prepare Cur/PVA/Collagen composite films (CPCF), which are shown in Figure 1(C). We also prepared PVA/Col films without loading curcumin by mixing collagen and PVA (Figure 1(B)).

### 3.2. Characteristics of cur/PCEC nanoparticles

The particle size and PDI of the prepared Cur/PCEC nanoparticles were measured and shown in Figure 1(D). The diameter and polydispersity of the Cur/PCEC nanoparticles were 43.63 ± 13.22 nm and 0.334 ± 0.403 nm, respectively. TEM revealed that the Cur/PCEC nanoparticles had a spherical regular structure (Figure 1(E)). Based on HPLC, the DL and EE were determined to be 9.61%±0.12% and 96.09%±1.21%, respectively.

### 3.3. *In vitro* drug release

As shown in Figure 1(F), the curcumin released from free curcumin, Cur/PCEC NPs, and CPCF was rapid in the first 12 h. Approximately 90% curcumin was released from Cur/PCEC NPs in a sustained manner within 120 h, while 76% curcumin was released from CPCF within 120 h. However, free curcumin exhibited a rapid release behavior and more than 98% curcumin was released into the medium within 48 h.

### 3.4. Antibacterial activity and *in vitro* cytotoxicity

**Figure 2.**
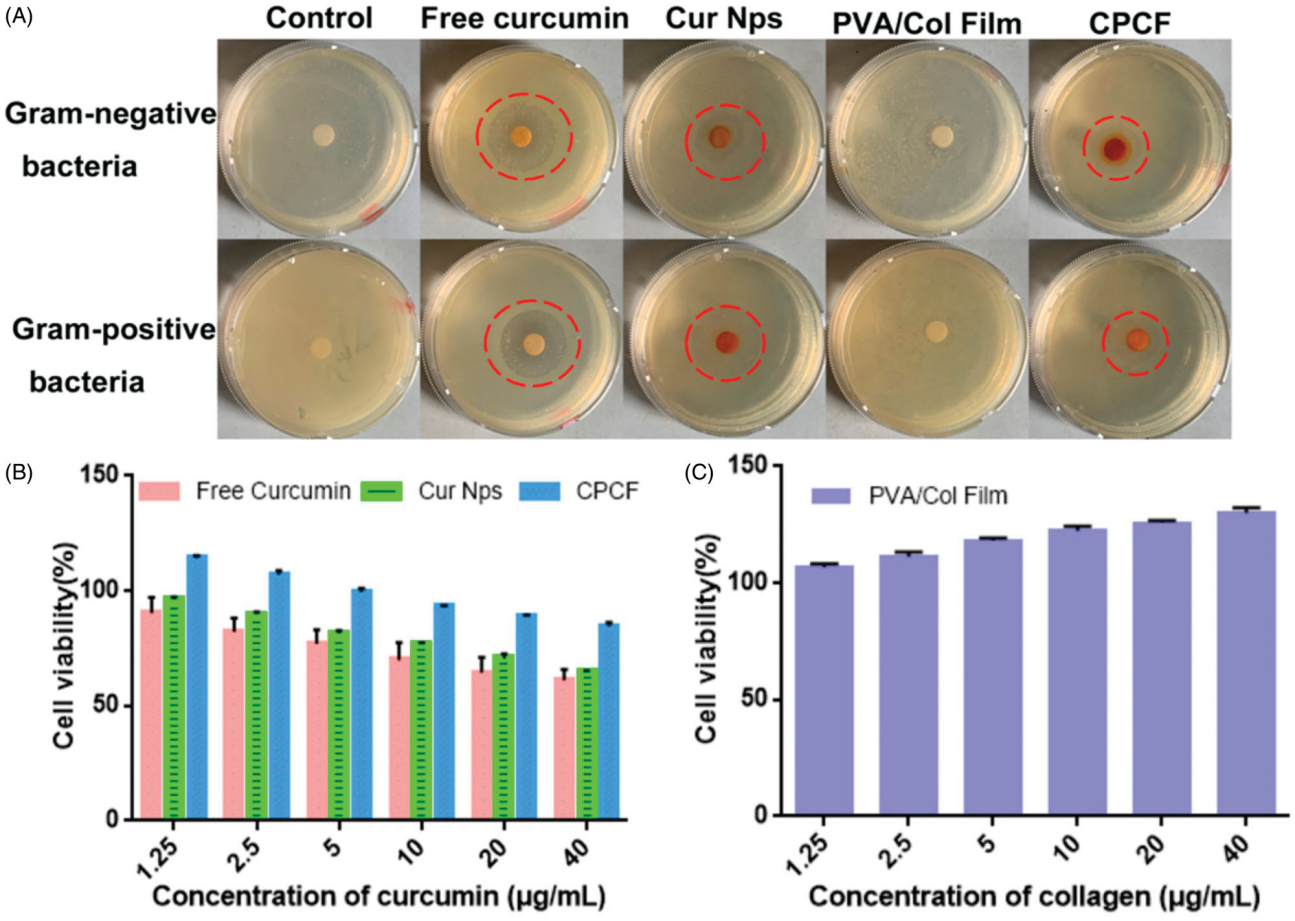
Antibacterial efficacy of free curcumin, Cur/PCEC NPs, PVA/Col film and CPCF (A); In vitro cytotoxicity of free curcumin, Cur/PCEC nanoparticles, CPCF (B) and PVA/Col film (C).

The antibacterial efficacies of free curcumin, Cur/PCEC nanoparticles, PVA/Col films, and CPCF were accessed and shown in Figure 2(A). A clear zone of inhibition was observed for both *S.aureus* and *E.coli* in groups of free curcumin, Cur/PCEC nanoparticles, and CPCF. No zones of inhibition were observed in the control and PVA/Col film groups.

To detect *in vitro* cytotoxicity of CPCF in human skin fibroblasts, an MTT assay was performed and the results are shown in Figure 2(B). It indicated that Cur/PCEC NPs reduced the toxic effects of curcumin and CPCF may have antagonized the toxic effects of curcumin. As seen in Figure 2(C), cell viability increased with an increase in collagen concentration.

### 3.5. *In vivo* wound healing study

**Figure 3.**
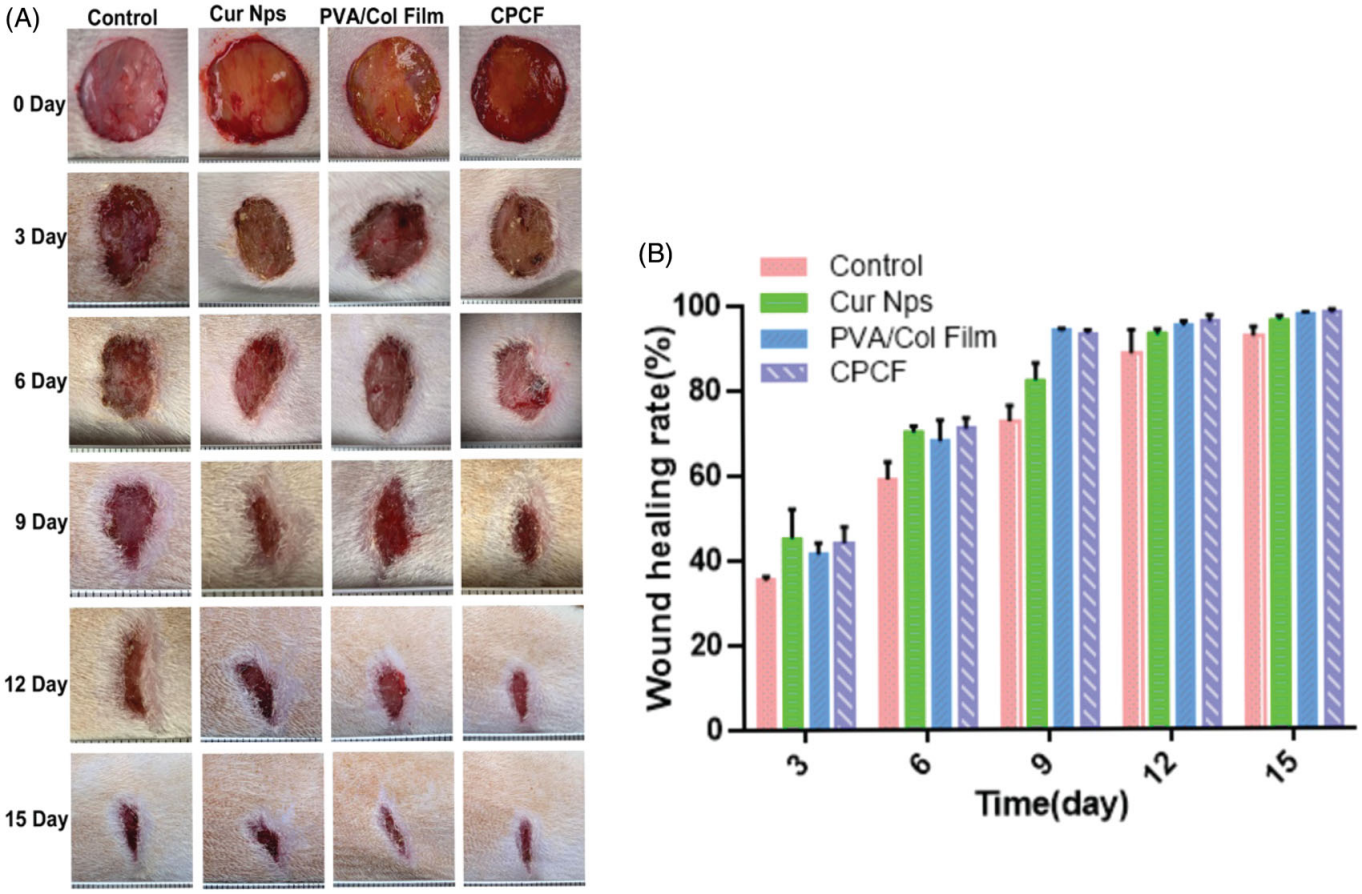
The pictures of wound surface healing over time in each group (A) and the percentage of wound closure versus healing time in each group (B) (*n* = 3).

To observe changes in wound area, wound images were captured as shown in Figure 3(A). On day 3 after surgery, the wound surfaces of animals in the Cur/PCEC NPs and CPCF groups had already dried and showed scab formation owing to the anti-inflammatory property of curcumin, while the wounds in the control and the PVA/Col film groups still exhibited some secretions. On day 6, some secretions could still be found on the wound surfaces in the control and PVA/Col film groups. On day 9, the wounds of all four groups appeared dry and did not appear infected; however, the wounds in the Cur/PCEC NPs and CPCF groups healed better than those in the other two groups. On the whole, the control group showed slower wound healing than the other three groups during the observation period. The wounds in the CPCF group healed better. The wound areas of mice were measured and the wound healing rate was calculated. As shown in Figure 3(B), the average wound healing rate on day 15 after surgery was 92.73%±1.83% in the control group, 96.40%±0.82% in the Cur/PCEC NPs group, 97.82%±0.39% in the PVA/Col film group, and 98.03%±0.79% in the CPCF group.

### 3.6. Histological examination

**Figure 4.**
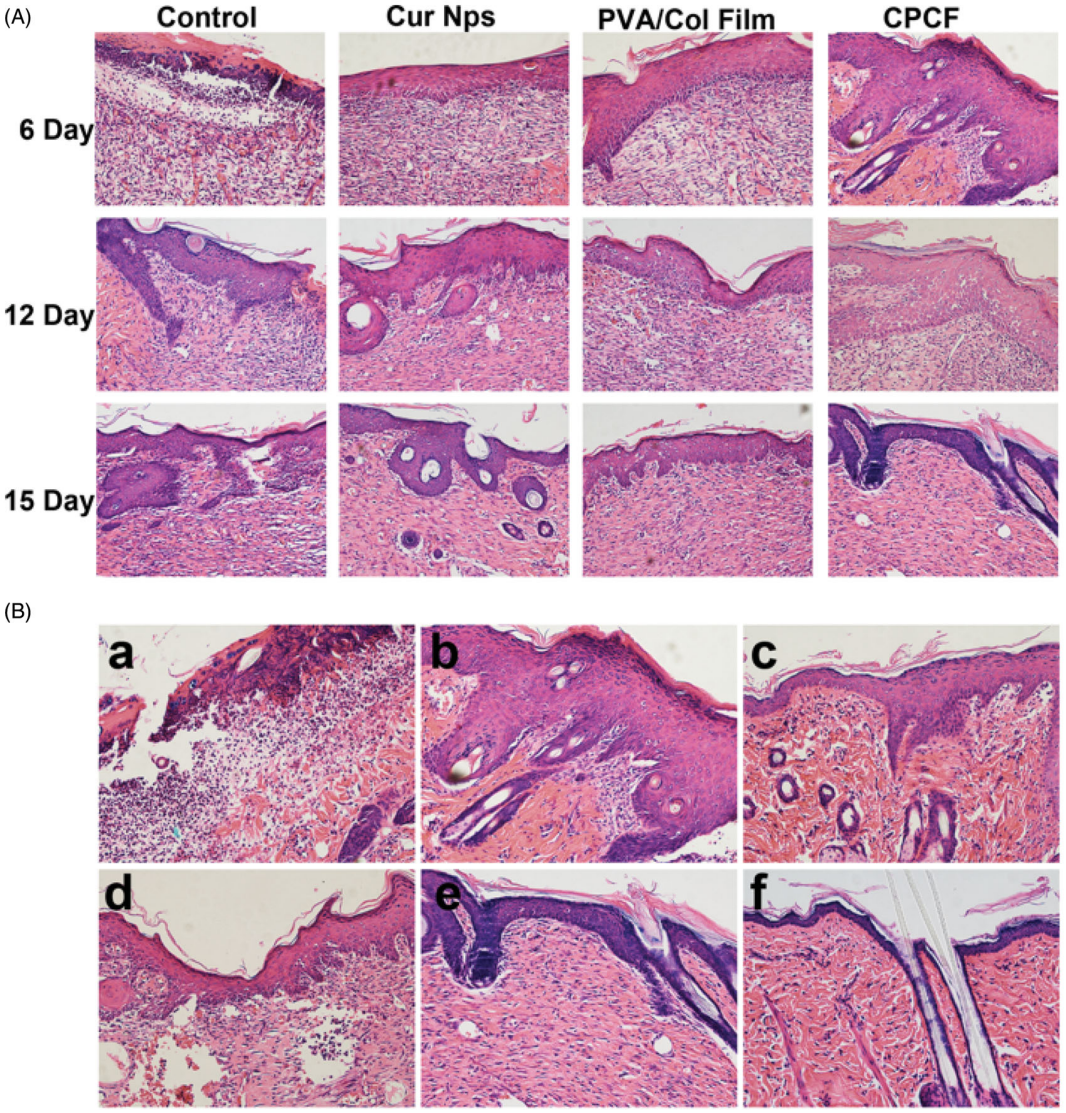
Representative H&E staining images of skin wounds. (A) The images of the control group, Cur Nps group, PVA/Col group and CPCF group on day 6, day 12 and day 15 after surgery (magnification × 200); (B) The images of the CPCF group wound on day 3 (a), day 6 (b), day 9 (c), day 12 (d), day 15 (e) and normal skin (f) (magnification × 200).

Figure 4(A) shows H&E staining images of issues at the skin wound sites from the four groups at different time points after surgery. On day 6, except for the control group, the skin at the wound sites in the other three groups was intact. There were large numbers of inflammatory cells in the control group and some granulation tissue was seen in all four groups. On day 12, there were still many inflammatory cells in the control and PVA/Col groups, but few inflammatory cells were seen in the Cur NPs and CPCF groups. On day 15, a clear dividing line was seen between the epidermis and the dermis in all groups and a clear cuticle was seen at the edge of the epidermis. The fibroblast arrangement in the control group was slightly disordered, while that in the other groups was more regular. Collagen fibers appeared denser, especially in the PVA/Col and CPCF groups. The Cur NPs group and CPCF group showed mature skin structure, but the CPCF group had higher skin maturity on day 15.

H&E staining images of the CPCF group on days 3, 6, 9, 12, and 15 shown in Figure 4(B) indicate a clear division between the epidermis and the dermis and in the appearance and distribution of hair follicles. On day 3, the local skin tissue appeared discontinuous with a large number of inflammatory cells with the presence of granulation tissue and partial fibroblasts. On day 6, there was a clear division between the epidermis and the dermis. Keratosis was visible at the edges of the epidermis. The skin in the wound area had been completely re-epithelialized, however, its structure was relatively loose. Several fibroblasts were seen in the dermis. On day 9, the epidermis resembled the normal skin dermis; fibroblasts that still showed a slightly disordered arrangement could be seen in the dermis. Additionally, blood vessels could also be seen. On day 12, the fibroblasts had arranged well and the epidermis was similar to that of the normal skin. On day 15, obvious hair follicles deep into the dermis were seen. The epidermis and the dermis appeared very similar to that of the normal skin.

**Figure 5.**
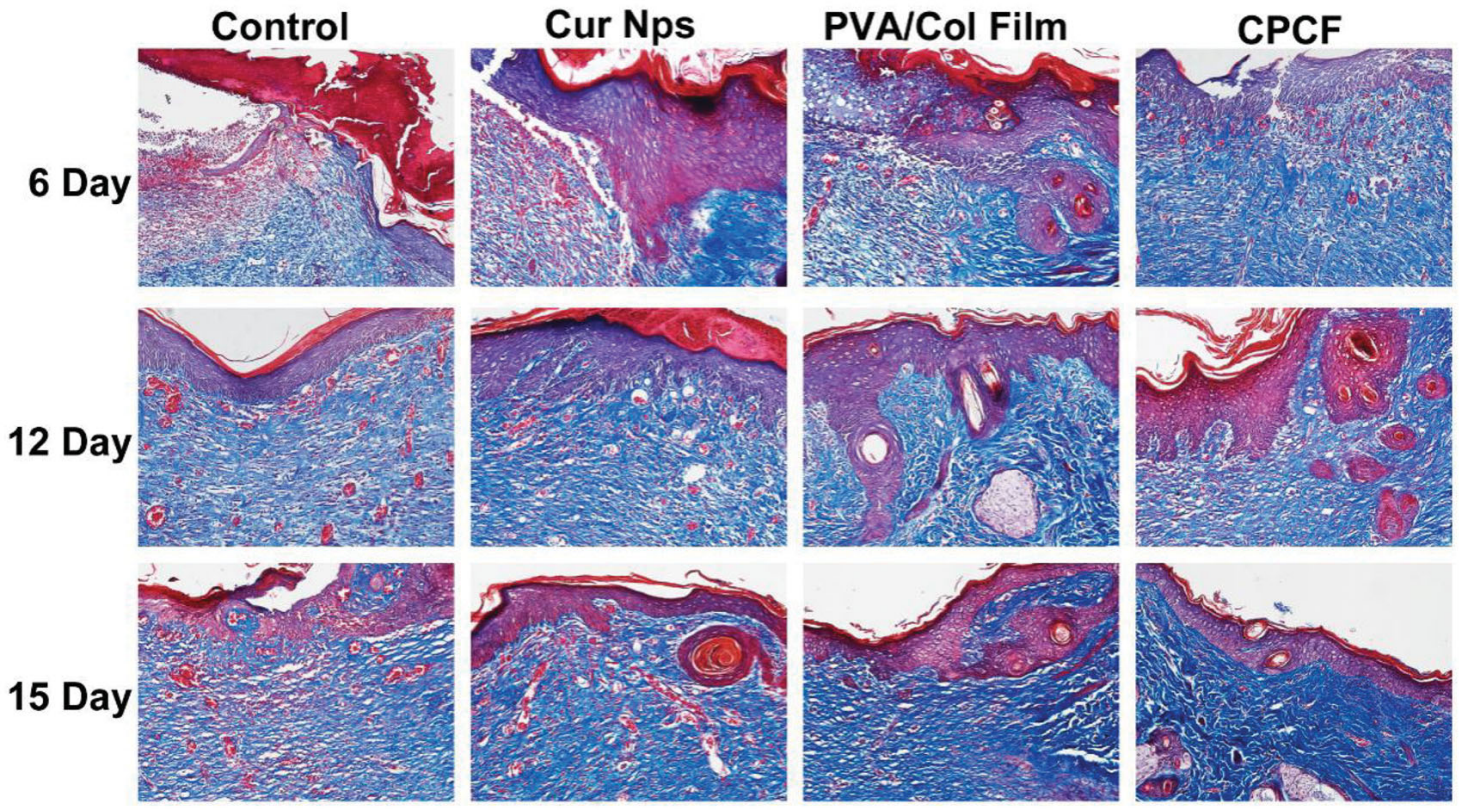
Masson’s trichrome staining of tissues in the group of the Control group, the Cur Nps group, the PVA/Col group and the CPCF group on day 6, day 12 and day 15 (magnification × 200).

Figure 5 shows the collagen fibers stained blue after Masson’s trichrome staining. On day 6, there was an increase in collagen fibers and blood vessels in the CPCF group. On day 12, the skin tissue and blood vessels could be seen intact in all groups. On day 15, large numbers of collagen fibers that were regularly arranged and densely distributed were observed in the CPCF group.

**Figure 6.**
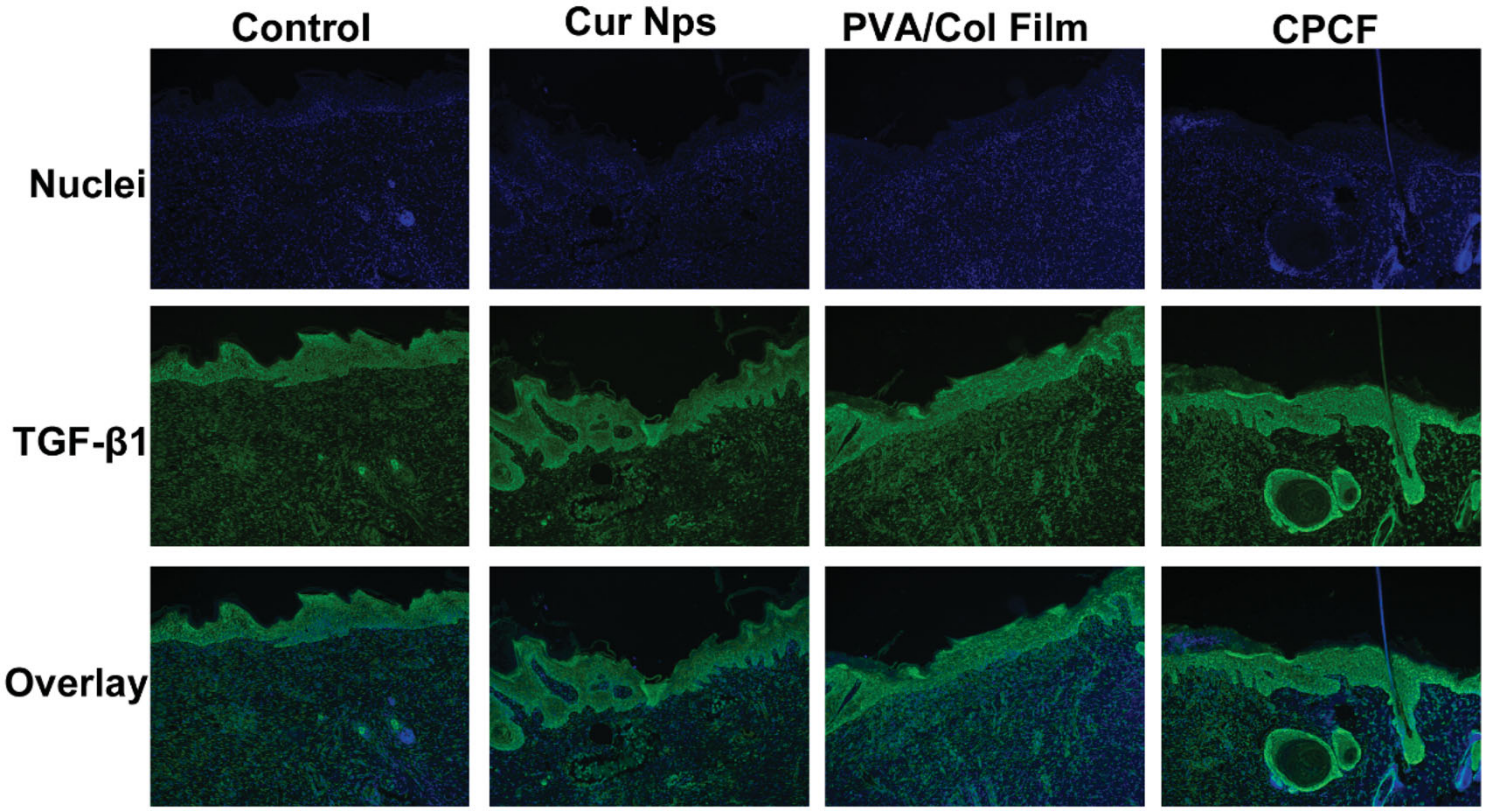
Images of immunofluorescence staining of TGF-β1 on day 12. Green fluorescence indicates TGF-β1, blue represents the nucleus (magnification × 200).

TGF-β1 is one of the newly discovered TGF-β superfamilies that regulate cell growth and differentiation. Figure 6 shows the fluorescence images of TGF-β1 expression on day 12. Green fluorescence indicates the expression of TGF-β1, while blue represents the nucleus. Based on the fluorescence signal, it was determined that TGF-β1 was highly expressed in all four groups, especially in the CPCF group. This result suggested that in the skin-repair and remodeling stages, the formation of TGF-β1 promoted the maturation of collagen fibers, and CPCF may have had an effect promoting the formation of TGF-β1.

## 4. Discussion

Wound healing is essential for postoperative recovery of skin function. Various wound dressings such as hydrogels, nanofibers, and films, have been widely investigated to promote wound healing. The major advantages of wound dressings include anti-infective and histocompatibility properties, among others (Albright et al., 2018; Augustine et al., 2019; He et al., 2019). To achieve the dual purpose of fighting infection and promoting healing, some antimicrobial strategies such as photothermal response or anti-infective drug curcumin are incorporated into skin dressings to inhibit bacterial proliferation at the wound site (Qu et al., 2018; Liang et al., 2020; Huang et al., 2020; Zhao et al., 2020). Different skin dressings have different advantages as well as disadvantages. For example, although hydrogels are convenient to use, especially in filling wounds of different shapes, theyisolate the wound from contact with oxygen in the air, which is not conducive to the proliferation and regeneration of cells and tissues at the wound site (Boucard et al., 2007). Although other fiber membranes in several studies use micropores to ensure the contact of the wound site with oxygen, it is often necessary to suture the composite membrane to the wound site to prevent it from falling off, which could, therefore, increase the difficulty of surgery (Murakami et al., 2010). To solve these problems, researchers have been exploring the composition and structure of skin dressings to improve the application potential of dressings. In this study, we developed a wound dressing, CPCF, comprising Cur/PCEC nanoparticles, collagen, and PVA. Similar to most wound dressings, CPCF also exhibited anti-infective properties. Its antibacterial effect is shown in Figure 2(A). A clear zone of inhibition around CPCF is seen; however, no zone of inhibition is present around the PVA/Col film for both Gram-positive and Gram-negative bacteria. This finding indicated that the antibacterial effect of CPCF could be attributed to curcumin, which is a well-known and effective antibacterial compound (Arunraj et al., 2019). In addition, owing to the slow-release effect of nanoparticles, CPCF released curcumin slowly and continuously, thus providing long-lasting protection during the wound-healing process (Figure 1(F)).

Apart from imparting anti-infective effects to skin dressing, it is also important to design the dressing such that its composition is similar to that of the skin. For example, collagen, as an important component of the skin, has received extensive attention in studies related to skin dressings. Many researchers incorporate it into skin dressings to enhance the biocompatibility of wound dressings and promote skin healing. Huan’s previous study demonstrates the potential of collagen as cell-culture substrates and cell-delivery vehicles (Chattopadhyay & Raines, 2014). Thus, in this study, we incorporated collagen type I into CPCF because it is an important component of the skin, more biocompatible compared to other natural polymers, and exhibits low antigenicity (Ramanathan et al., 2020). Results from the MTT assay revealed that CPCF had lower cytotoxicity than that of free curcumin and Cur/PCEC nanoparticles, which are consistent with those from other studies (Hu et al., 2017; Yang et al., 2019). Results from Masson’s trichrome staining (Figure 5) revealed that the new skin in animals of the CPCF group had denser collagen fibers than that of the other groups. Our findings also confirmed that the incorporation of collagen increased the biocompatibility of CPCF, promoted fibroblasts adhesion and proliferation, and further enhanced wound healing.

Although collagen has received extensive attention in the research of skin dressings, it is rarely used directly as a dressing matrix. This is because collagen has poor strength and its inherent film-formation abilities are not satisfactory. Therefore, other components are often added to enhance these properties. For example, chitosan and PLLA are used as fillers to enhance the film-forming properties of collagen. In the current study, we used PVA as a filler owing to its biocompatibility and film-forming properties. PVA has been used in implants, artificial organs, contact lenses, fibers, films, etc., because it is water soluble, nontoxic, biocompatible, and exhibits desirable chemical and mechanical strength (Niranjan et al., 2019). And Seyed Mahdi Saeed’s research demonstrates that PVA can absorb exudates (Saeed et al., 2017) and help the composite film fit the wound closely.

In this study, we constructed a dressing with good biocompatibility and antibacterial efficacy, and the *in vivo* and *in vitro* results confirmed our hypothesis. The incorporation of PVA improved the film-forming properties of collagen. The complete film could be satisfactorily attached to the wound surface. During the observation period, no wound discharge, redness, swelling, or purulent phenomena occurred, which indicated the anti-infective effect of the film. *In vitro* antibacterial and drug-release tests proved that CPCF-loaded curcumin nanoparticles had similar sustained drug-release behavior and significant antibacterial activity. The composite membrane did not show obvious cytotoxicity, and even with an increase in collagen, there was an increase in cell proliferation. H&E staining confirmed that the inflammatory cells in the wound tissue of the CPCF group were the least on day 6, indicating that our composite membrane had good biocompatibility and anti-inflammatory effects. Results from Masson’s trichrome staining revealed an increase in the collagen fibers and blood vessels in the CPCF group on day 6, which also indicated the biocompatibility of CPCF. TGF-β1, a newly discovered member of the TGF-β superfamily, appears to significantly promote keratinocyte proliferation and epidermal layer remodeling and regeneration (Chong et al., 2020). In our study, TGF-β1 was highly expressed in the CPCF group than the other groups. This result further confirms that CPCF has good biocompatibility and can promote wound healing. In addition, *in vivo* wound healing experiments proved that wounds in the CPCF group healed faster than those in the other groups. Therefore, CPCF may show promise in wound dressing and skin tissue engineering.

## 5. Conclusion

In this study, CPCF containing curcumin nanoparticles, collagen, and PVA was prepared as a dermal dressing to enhance wound healing. Curcumin nanoparticles not only improved the water solubility of curcumin but also provided CPCF with long-term protection and treatment for skin wounds owing to its sustained drug-release effect. The CPCF demonstrated superior performance as an antibacterial and exhibited histocompatibility. Results of our *in vivo* evaluation revealed that the CPCF was suitable for wound management and promoted wound healing. Therefore, CPCF may be used as an effective wound dressing and also shows potential in skin tissue engineering.
